# Molecular risk assessment of BIG 1-98 participants by expression profiling using RNA from archival tissue

**DOI:** 10.1186/1471-2407-10-37

**Published:** 2010-02-09

**Authors:** Janine Antonov, Vlad Popovici, Mauro Delorenzi, Pratyaksha Wirapati, Anna Baltzer, Andrea Oberli, Beat Thürlimann, Anita Giobbie-Hurder, Giuseppe Viale, Hans Jörg Altermatt, Stefan Aebi, Rolf Jaggi

**Affiliations:** 1 Department of Clinical Research, University of Bern, Bern, Switzerland; 2 National Center of Competence in Research (NCCR) Molecular Oncology, Swiss Institute of Bioinformatics (SIB), Lausanne, Switzerland; 3 Senology Center of Eastern Switzerland, Kantonsspital, St. Gallen, Switzerland; 4 International Breast Cancer Study Group Statistical Center, Dana-Farber Cancer Institute, Boston, MA, USA; 5 Division of Pathology and Laboratory Medicine, European Institute of Oncology, University of Milan, Milan, Italy; 6 Pathology Länggasse, Bern, Switzerland; 7 Medical Oncology, University Hospital Bern, Bern, Switzerland; 8 Swiss Group of Clinical Cancer Research (SAKK), Bern, Switzerland

## Abstract

**Background:**

The purpose of the work reported here is to test reliable molecular profiles using routinely processed formalin-fixed paraffin-embedded (FFPE) tissues from participants of the clinical trial BIG 1-98 with a median follow-up of 60 months.

**Methods:**

RNA from fresh frozen (FF) and FFPE tumor samples of 82 patients were used for quality control, and independent FFPE tissues of 342 postmenopausal participants of BIG 1-98 with ER-positive cancer were analyzed by measuring prospectively selected genes and computing scores representing the functions of the estrogen receptor (eight genes, ER_8), the progesterone receptor (five genes, PGR_5), Her2 (two genes, HER2_2), and proliferation (ten genes, PRO_10) by quantitative reverse transcription PCR (qRT-PCR) on TaqMan Low Density Arrays. Molecular scores were computed for each category and ER_8, PGR_5, HER2_2, and PRO_10 scores were combined into a RISK_25 score.

**Results:**

Pearson correlation coefficients between FF- and FFPE-derived scores were at least 0.94 and high concordance was observed between molecular scores and immunohistochemical data. The HER2_2, PGR_5, PRO_10 and RISK_25 scores were significant predictors of disease free-survival (DFS) in univariate Cox proportional hazard regression. PRO_10 and RISK_25 scores predicted DFS in patients with histological grade II breast cancer and in lymph node positive disease. The PRO_10 and PGR_5 scores were independent predictors of DFS in multivariate Cox regression models incorporating clinical risk indicators; PRO_10 outperformed Ki-67 labeling index in multivariate Cox proportional hazard analyses.

**Conclusions:**

Scores representing the endocrine responsiveness and proliferation status of breast cancers were developed from gene expression analyses based on RNA derived from FFPE tissues. The validation of the molecular scores with tumor samples of participants of the BIG 1-98 trial demonstrates that such scores can serve as independent prognostic factors to estimate disease free survival (DFS) in postmenopausal patients with estrogen receptor positive breast cancer.

**Trial Registration:**

Current Controlled Trials: NCT00004205

## Background

Clinical and histopathological factors such as lymph node status, tumor size, histological grade, age, and expression of estrogen receptor (ER) and Her2 have traditionally guided treatment decisions of patients with operable breast cancer [1,2]. Various prognostic models are based on these factors, for example the Nottingham Prognostic Index (NPI) [3,4], Adjuvant!Online [5,6] and others [7]. Despite providing excellent estimates of the average risk of recurrence, there remains substantial variation in outcome which may be explained by molecular differences among these tumors [8,9].

DNA-chip based expression analyses have confirmed the heterogeneity of breast cancer and allowed the development of clinically relevant gene "signatures" or "profiles" [10-20]. Such profiles are being implemented widely in routine patient care even though many signatures were developed and validated on heterogeneous patient cohorts with respect to stage of disease and therapy. The utility of gene signatures as part of the decision making process is being validated in ongoing studies (TAILORx [21] and MINDACT [22]). Most profiling studies are based on fresh-frozen (FF) or RNAlater conserved tissue. Such material must be collected and processed separately after surgery, complicating the implementation of molecular analyses into the clinical workflow. Procedures based on formalin-fixed, paraffin-embedded (FFPE) material simplify the acquisition of tumor material and can easily be established as part of the routine pathological procedures. In addition, FFPE tissues collected in the framework of clinical trials could be a valuable resource for future research.

We prospectively selected genes from publicly available microarray data and developed molecular scores representing the ER, progesterone receptor (PgR), Her2 and proliferation (PRO) status, and the overall risk of recurrence (RISK). The reproducibility and robustness of the molecular scores was validated by comparing expression data with RNA from FF and FFPE material of 82 tumors. Molecular scores were determined from 342 ER positive tumor samples of the BIG 1-98 clinical trial. Multivariate Cox proportional hazard models revealed that molecular scores are independent prognostic factors to estimate disease free survival (DFS).

## Methods

To assess the quality of expression profiling from FFPE material, matched FF and FFPE samples from 82 human breast cancers were used. Histopathological information was irreversibly anonymized according to Swiss law. Independent FFPE blocks and corresponding clinical data of 437 Swiss participants of the trial BIG 1-98 were provided by the International Breast Cancer Study Group. The ethics committees and required health authorities of each participating institution approved the study protocol, and all patients gave written informed consent (ClinicalTrials.gov number, NCT00004205) [23]. Retrospective tissue collection was carried out in accordance with institutional guidelines and national laws. The patient and tumor characteristics of these patients were similar to the entire BIG 1-98 population (Table 1). BIG 1-98 is a randomized controlled clinical trial of adjuvant hormonal therapy for postmenopausal patients with endocrine-responsive breast cancer comparing 4 arms: 5 years of tamoxifen, 5 years of letrozole, two years of tamoxifen followed by 3 years of letrozole, or vice versa [24-26]. All the patients from the BIG 1-98 were treated by mastectomy or breast conserving surgery [24-26]. The available paraffin blocks contained material derived from representative tumor regions.

**Table 1:** Gene Identifications, Categories and Score affiliations

Gene	Category	**Accession Nr**.	Description	AS	Score
GUSB	Control	NM_000181.1	glucuronidase, beta	81	control
RPLP0	Control	NM_053275.3 NM_001002.3	ribosomal protein, large, P0	105	control
UBB	Control	NM_018955.2	ubiquitin B	120	control
AR	ER	NM_001011645.1 NM_000044.2	androgen receptor (dihydrotestosterone receptor; testicular feminization; spinal and bulbar muscular atrophy; Kennedy disease)	72	ER_8
ERBB4	ER	NM_001042599.1 NM_005235.2	v-erb-a erythroblastic leukemia viral oncogene homolog 4 (avian)	77	ER_8
ESR1	ER	NM_000125.2	estrogen receptor 1	62	ER_8 ER_4
FOXA1	ER	NM_004496.2	forkhead box A1	74	ER_8
GATA3	ER	NM_001002295.1 NM_002051.2	GATA binding protein 3	80	ER_8
MAPT	ER	NM_016834.2 NM_016835.2 NM_016841.2 NM_005910.3	microtubule-associated protein tau	60	ER_8
MYB	ER	NM_005375.2	v-myb myeloblastosis viral oncogene homolog (avian)	96	ER_8
XBP1	ER	NM_005080.2	X-box binding protein 1	60	ER_8
BCL2	ER	NM_000633.2	B-cell CLL/lymphoma 2	81	ER_4
GREB1	PGR	NM_033090.1 NM_148903.1 NM_014668.2	GREB1 protein	77	PGR_5
PGR	PGR	NM_000926.3	progesterone receptor	118	PGR_5 ER_4
RAB31	PGR	NM_006868.2	RAB31, member RAS oncogene family	109	PGR_5
RBBP8	PGR	NM_203291.1 NM_203292.1 NM_002894.2	retinoblastoma binding protein 8	75	PGR_5
SERPINA3	PGR	NM_001085.4	serpin peptidase inhibitor, clade A (alpha-1 antiproteinase, antitrypsin), member 3	70	PGR_5
SCUBE2	PGR	NM_020974.1	CEGP1, signal peptide, CUB domain, EGF-like 2	64	ER_4
ERBB2	HER2	NM_001005862.1 NM_004448.2	v-erb-b2 erythroblastic leukemia viral oncogene homolog 2, neuro/glioblastoma derived oncogene homolog (avian)	120	HER2_2
GRB7	HER2	NM_005310.2	growth factor receptor-bound protein 7	70	HER2_2
CCNB2	Proliferation	NM_004701.2	cyclin B2	73	PRO_10
CCNE2	Proliferation	NM_057735.1 NM_057749.1	cyclin E2	70	PRO_10
CDC2	Proliferation	NM_033379.2 NM_001786.2	cell division cycle 2, G1 to S and G2 to M	92	PRO_10
CENPF	Proliferation	NM_016343.3	centromere protein F, 350/400 ka (mitosin)	99	PRO_10
KIF20A	Proliferation	NM_005733.1	kinesin family member 20A	130	PRO_10
MKI67	Proliferation	NM_002417.3	antigen identified by monoclonal antibody Ki-67	131	PRO_10 PRO_5
ORC6L	Proliferation	NM_014321.2	origin recognition complex, subunit 6 like (yeast)	78	PRO_10
PRC1	Proliferation	NM_199413.1 NM_199414.1 NM_003981.2	protein regulator of cytokinesis 1	66	PRO_10
SPAG5	Proliferation	NM_006461.3	sperm associated antigen 5	114	PRO_10
TOP2A	Proliferation	NM_001067.2	topoisomerase (DNA) II alpha 170 kDa	125	PRO_10
AURKA	Proliferation	NM_003600.2	STK15 aurora kinase A	85	PRO_5
BIRC5	Proliferation	NM_001012271.1 NM_001168.2	baculoviral IAP repeat-containing 5 (survivin)	93	PRO_5
CCNB1	Proliferation	NM_031966.2	cyclin B1	104	PRO_5
MYBL2	Proliferation	NM_002466.2	v-myb myeloblastosis viral oncogene homolog (avian)-like 2	81	PRO_5

Abbreviation: AS, amplicon size

### Tissue samples and data processing

The RNA was isolated from 4 sections (25 μm) of FF material and from 10 paraffin sections (10 μm thick) as described previously [27]. After demodification, the RNA was bound to silica-based columns, DNase I digested and eluted with water. The protocols and reagents for RNA isolation from FF and FFPE tissues were recently incorporated in commercial protocols (RNAready and FFPE RNAready, AmpTec, Hamburg, Germany). RNA qualities were assessed on an Agilent 2100 Bioanalyzer (Agilent Technologies, Inc., Santa Clara, CA, USA). RNA prepared from FF material had a RIN>6 (RNA integrity number), the RIN of RNA from FFPE was 2-3. The percentage of tumor cells in each FFPE block was evaluated on stained tissue sections. From 437 available FFPE samples 43 samples (9.8%) with less than ~30% tumor cells, 10 ER-negative tumor samples and 7 samples (1.6%) with less than 1.5 μg total RNA recovery were excluded from further analysis. Approximately 30% of the sections contained 30-50% tumor cells, and about 60% contained 50-100% tumor cells. Each of the remaining RNAs was tested by quantitative reverse transcription PCR (qRT-PCR) with 3 control genes (GUSB, RPLP0 and UBB). The mean of the three raw Cts (cycle thresholds) was determined. In 35 samples (8%) the mean Ct was >31, indicating poor quality of the RNA. These RNAs were excluded from further analyses. For the remaining 342 RNAs (78.3%), the expression of 34 genes (see Table 1) was measured by qRT-PCR on TaqMan Low Density Arrays (TLDAs) (Applied Biosystems, Foster City, CA, USA) using a one step protocol (Invitrogen, Basel, Switzerland) on an Applied Biosystems 7900HT instrument. Technical replicates were performed for several intact and several partially degraded RNAs from FF and FFPE material, respectively. They revealed Pearson correlation coefficients higher than 0.95 for all 34 assays.

Genes with high correlation to the expression of ER, PgR, Her2 and proliferation related genes were prospectively selected from publicly available microarray data [28]. A complete list of microarray data sets used in the meta-analysis is available at ".http://breast-cancer-research.com/content/10/4/R65/table/T1[28] (Additional File 1, Table S1). The scores were defined by giving equal weight to each gene in the four groups (proliferation, estrogen response, progesterone response, Her2 response). Thus, a training set was not used as the scores were based on in silico gene selection.

Raw Ct values were normalized against the mean expression of GUSB, RPLP0 and UBB. Scores for ER (ER_8), PgR (PGR_5), Her2 (HER2_2) and proliferation (PRO_10) were defined as mean expression of all genes in each category (Table 1). A RISK score comprising 25 genes was calculated as follows: RISK_25 = PRO_10+HER2_2-(8 × ER_8+5 × PGR_5)/13. For comparison, ER_4 and PRO_5 scores were calculated based on 4 and 5 genes described previously [27]. The genes corresponding to ER_4 and PRO_5 scores corresponded to the genes used for calculating the recurrence score (RS) [29].

### Concordance of molecular scores and pathological parameters

Histopathological data of BIG 1-98 samples were derived from a central review, with the exception of the grade which was locally assessed. The ER and PgR status were dichotomized into positive (≥ 10% immunoreactive cells) or negative (<10%) [30]. Her2 was measured by fluorescence in-situ hybridization or immunohistochemistry (IHC) and tumors were classified according to Rasmussen et al. [31]. The Ki-67 labeling index (LI) was centrally assessed by IHC as described and classified into low or high using the median LI (11%) as cut-off [32]. The same assays and cut-offs were used for the 82 matched samples with the exception of Her2 which was measured using the CB11 monoclonal antibody and using a cut-off of ≥ 50% [33]. Continuous molecular scores were compared to binary IHC parameters using the area under the curve (AUC). The 95% confidence intervals (CI) were estimated by a bootstrap method (100 bootstraps). Two-sided Mann-Whitney tests were used to assess the association between clinicopathological factors and scores.

### Statistical analyses

Primary endpoint of survival analyses was DFS as defined previously [25]. Forty-five events were observed in 342 patients with a median follow-up time (estimated by reverse Kaplan-Meier [34]) of 60 months. DFS was estimated by Kaplan Meier analysis. Patients were classified into low and high PRO or RISK scores using the corresponding median score as cut-off. The differences in survival experience between the two resulting groups were assessed with log rank tests. Univariate and multivariate Cox proportional hazard models were used [35] and hazard ratios (HR), CIs and p-values were obtained. The multivariate models were assessed using the log-likelihood and the deviance of residuals. Likelihood ratio tests (LRT) were used to compare different nested multivariate models. No adjustments were made for multiple testing. Univariate Cox proportional hazard models were applied to estimate the rate of events and to produce corresponding plots.

## Results

### Reliable expression profiling from FFPE tumor tissue

Gene expression was measured from 34 genes using TLDAs with RNA isolated from FF and FFPE material of 82 breast cancers. These data were used solely for the assessment of the expression profiling from FFPE material. Pearson correlation coefficients between FF and FFPE expression values for each tumor and all assays ranged from 0.91 to 0.98. The mean increase of raw Ct values derived of FFPE compared to matched FF tissues was 1.30 units. This Ct shift was mostly compensated by normalization (Additional File 2, Figure S1. and Additional File 3, Figure S2).

**Figure 1:**
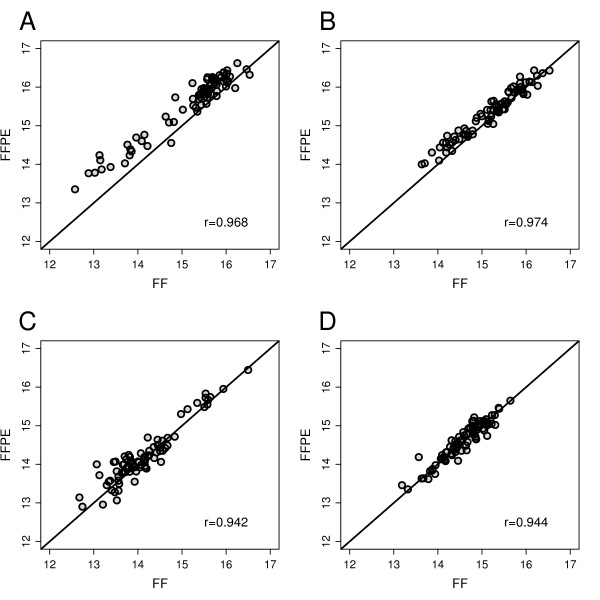
**Comparison of scores computed from intact RNA and partially degraded RNA from FFPE material**. Scores were determined for RNA from FF material and RNA from corresponding FFPE tumor material of 82 patients. Scatter plots are shown between scores from FF and FFPE tissues representing ER_8 (A), PGR_5 (B), HER2_2 (C) and PRO_10 (D) for each tumor. Pearson correlations are indicated.

Unsupervised hierarchical clustering demonstrated the stability of gene clusters and revealed an excellent agreement between FF- and FFPE-based expression profiles (Additional File 4, Figure S3). Molecular scores were determined for ER, PGR, HER2 and PRO. A linear relationship of scores was found for RNA from FF and RNA from FFPE material (Figure 1). Pearson correlation coefficients for the four scores were 0.968, 0.974, 0.942 and 0.944, respectively. The distributions of ER_8, PGR_5 and HER2_2 scores are shown as histograms together with the fitted mixture of two Gaussian distributions (Additional File 1, Figure S4) used for discriminating the subtypes.

The agreement between molecular scores and corresponding binary IHC variables was assessed by receiver operating characteristic (ROC) curves and AUC. AUCs and 95% CI were calculated for ER_8 (FF = 0.940 (0.835-1.00), FFPE = 0.931 (0.804-1.00)), PGR_5 (FF = 0.919 (0.828-0.986), FFPE = 0.916 (0.806-0.987) and HER2_2 (FF = 0.961 (0.895-1.00), FFPE = 0.963 (0.915-0.993)). PRO_10 was compared with IHC data for Ki-67 using a cut-off of 11% and the resulting AUCs were 0.798 (0.609-0.900) for FF and 0.810 (0.660-0.907) for FFPE, respectively. In conclusion, the agreement of the IHC with FFPE samples was as good as with FF samples.

### Concordance between pathological parameters and molecular scores for tumors of the BIG 1-98 clinical trial

Molecular scoring was applied to an independent set of tissue samples from Swiss patients participating in the BIG 1-98 randomized clinical trial and scores were compared to centrally assessed histopathological data by ROC curves. From a total of 437 provided tumor samples 342 ER-positive tumors (78.3%) were suitable for analysis. The AUC was 0.974 (95% CI = 0.946-0.995) for HER2_2 and 0.847 (95% CI = 0.794-0.902) for PGR_5. PRO_10 scores positively correlated with Ki-67 LI (Pearson correlation coefficient 0.51); the AUC was 0.815 (95% CI = 0.768-0.864) for Ki-67 binarized at 11% [32].

### The PRO_10 score correlates with histological grade and other clinical factors

**Figure 2:**
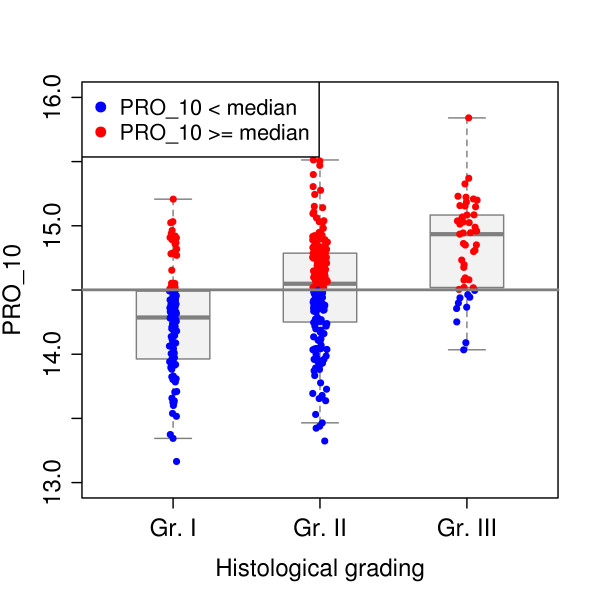
**Comparison of scores and immunohistochemical analysis**. Correlation of histological grading and PRO_10 score. The 342 tumors were classified according to histological grading. The data are shown as boxplots with median (solid line), interquartile ranges (boxes) and minimum and maximum non-outlier values (whiskers). The PRO_10 scores higher and lower than the median are indicated as red and blue dots, respectively for each grade.

The histological grade was assessed according to Elston and Ellis [36]. The PRO_10 score positively correlated with Elston and Ellis scores and with grade (Pearson correlation coefficient 0.453 and 0.409, respectively) (Figure 2). Furthermore, PRO_10 scores were significantly higher in Her2 positive tumors, in tumors larger than 2 cm and in tumors with axillary lymph node metastasis as compared to Her2 negative tumors, T1 tumors and N0 tumors (p ≤ 0.0015, Mann-Whitney tests), respectively (data not shown).

### PRO and RISK scores predict disease free survival in lymph node positive patients and patients with grade II breast cancer

**Figure 3:**
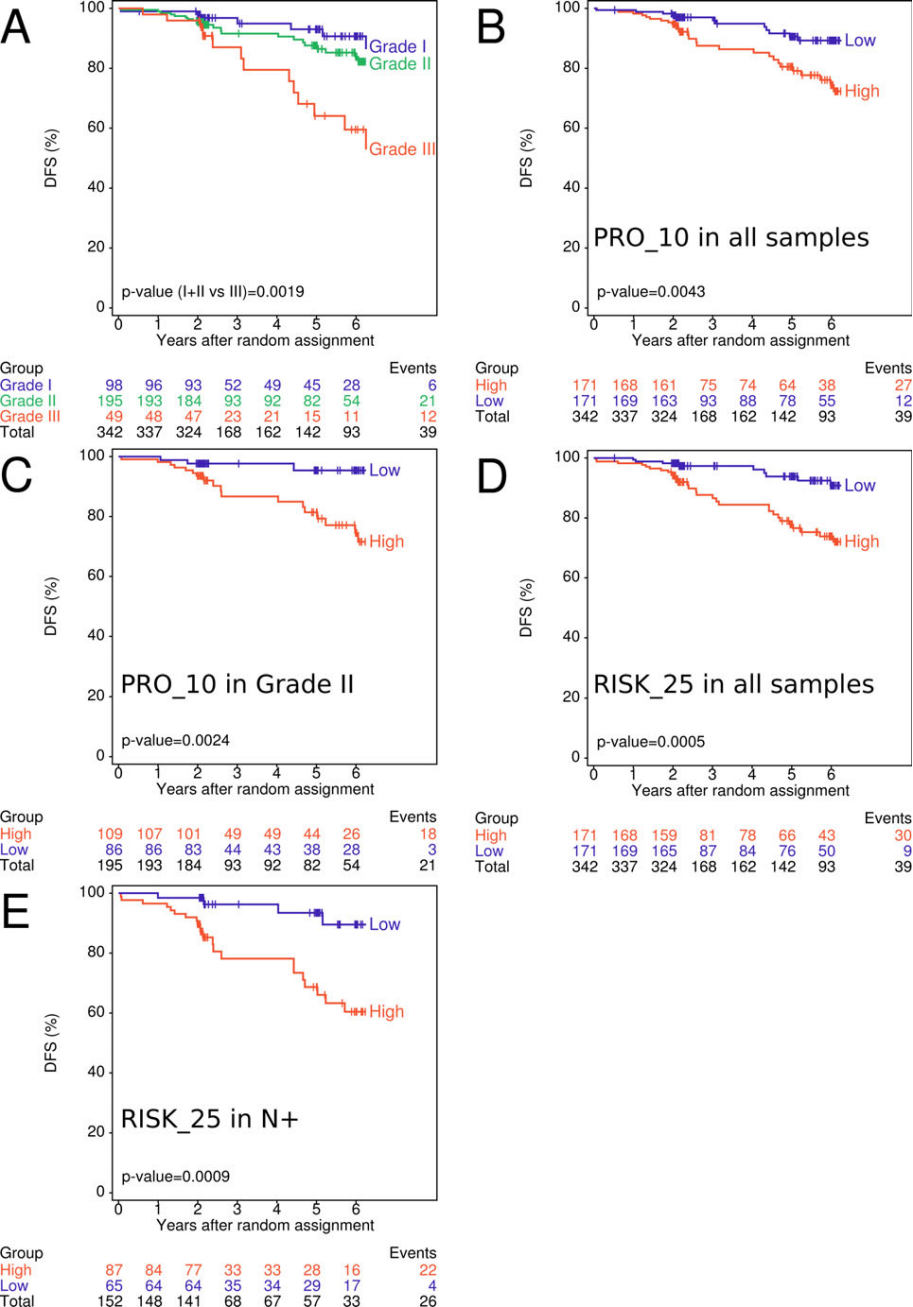
**Survival data based on molecular scores**. Kaplan-Meier plots for DFS. Patients were stratified into grade I (blue), II (green) and III (red line) (A), into low (blue) and high (red) PRO_10 scores in all samples (B) and in Grade II samples (C). The RISK_25 score is shown for all samples (D) and for tumors of patients with lymph node positive (N+) cancer (E). Median values of the scores were used as cut-offs. The p-values correspond to Log-rank test.

The prognostic values of PRO_10 and RISK_25 scores were assessed by their ability to assign patients to low and high risk groups. Patients were stratified according to histological grade and low or high PRO_10 and RISK_25 scores using the corresponding medians as cut-offs (Figure 3). As expected, patients with grade III tumors had poorer DFS than patients with grade I or grade II tumors (p = 0.0019, panel A). High PRO_10 scores correlated with poorer DFS compared to low scores in all (p = 0.0043, panel B) and in histological grade II tumors (p = 0.0024, panel C). Similarly, RISK_25 discriminated between favorable and poor DFS in all (p = 0.0005, panel D) and in node positive tumors (p = 0.0009, panel E). Univariate Cox proportional hazards regression analysis confirmed these observations.

The PGR_5, PRO_10 and RISK_25 scores were all significant predictors of DFS (p < 0.05) as were histological grade, tumor size, number of positive lymph nodes and Ki-67 LI (Table 2). The PRO_5 score was also a significant predictor of DFS but PRO_10 score was numerically better than PRO_5 in terms of log-likelihood (L) and deviance of residuals (D) (PRO_10: L = -223.35, D = 225.83; PRO_5: L = -224.16, D = 227.57).

**Table 2:** Baseline characteristics.

Characteristic	Patients with FFPE profiles from Swiss participants used in the study (N = 342)	Provided material of Swiss participants (N = 437)	Patients of the BIG 1-98 population not used in the study (N = 7573)	Overall BIG 1-98 population (N = 8010)
Menopausal category - N (%)				
Postmen. before chemo	321 (93.9)	413 (94.5)	7279 (96.1)	7692 (96.0)
Postmen. after chemo	10 (2.9)	11 (2.5)	181 (2.4)	192 (2.4)
Premenopausal (ineligible)	0 (0.0)	2 (0.5)	21 (0.3)	23 (0.3)
Uncertain status	10 (2.9)	10 (2.3)	92 (1.2)	102 (1.3)
Unknown/missing	1 (0.3)	1 (0.2)	0	1 (<0.1)

Age at randomization - years				
Median	62	62	61	61
Range	41-86	41-86	38-90	38-90

Tumor size - N (%)				
≤ 2 cm	195 (57.0)	251 (57.4)	4706 (62.1)	4957 (61.9)
> 2 cm	144 (42.1)	179 (41.0)	2794 (36.9)	2973 (37.1)
Unknown/missing	3 (0.9)	7 (1.6)	73 (1.0)	80 (1.0)

Tumor grade - N (%)				
Grade 1	94 (27.5)	124 (28.4)	2007 (26.5)	2131 (26.6)
Grade 2	196 (57.3)	251 (57.4)	3649 (48.2)	3900 (38.7)
Grade 3	49 (14.3)	59 (13.5)	1166 (15.4)	1225 (15.3)
Unknown/missing	3 (0.9)	3 (0.7)	751 (9.9)	754 (9.4)

Nodal status - N (%)				
Negative (including Nx)	186 (54.4)	245 (56.1)	4342 (57.3)	4587 (57.3)
Positive	152 (44.4)	188 (43.0)	3123 (41.2)	3311 (41.3)
Unknown/missing	4 (1.2)	4 (1.0)	108 (1.4)	112 (1.4)

ER and PgR status - N (%)				
ER pos and PgR pos.	268 (78.4)	340 (77.8)	4715 (62.3)	5055 (63.1)
ER pos and PgR neg.	66 (19.3)	87 (19.9)	1544 (20.4)	1631 (20.4)
ER pos and PgR unknown	1 (0.3)	1 (0.2)	1153 (15.2)	1154 (14.4)
ER neg and PgR pos.	5 (1.5)	7 (1.6)	136 (1.8)	143 (1.8)
ER unknown, PGR pos.	0	0	7 (0.1)	7 (0.1)
Other	2 (0.6)	2 (0.5)	18 (0.3)	20 (0.2)

Local therapy - N (%)				
BCS and RT	236 (69.0)	310 (70.9)	3987 (52.7)	4297 (53.7)
BCS and no RT	13 (3.8)	16 (3.7)	228 (3.0)	244 (3.0)
Mastectomy and RT	24 (7.0)	25 (5.7)	1415 (18.7)	1440 (18.0)
Mastectomy and no RT.	68 (19.9)	85 (19.5)	1926 (25.4)	2011 (25.1)
Other	1 (0.3)	1 (0.2)	17 (0.2)	18 (0.2)

Adjuvant or neoadjuvant				
chemo (or both) - N (%)				
Yes	133 (38.9)	159 (36.4)	1865 (24.6)	2024 (25.3)
No	209 (61.1)	278 (63.6)	5708 (75.4)	5986 (74.7)

Abbreviations: BCS, breast conserving surgery; Nx, nodal status unknown; postmen., postmenopausal; RT, radiotherapy; PgR, progesterone receptor; pos., positive; neg., negative

**Figure 4:**
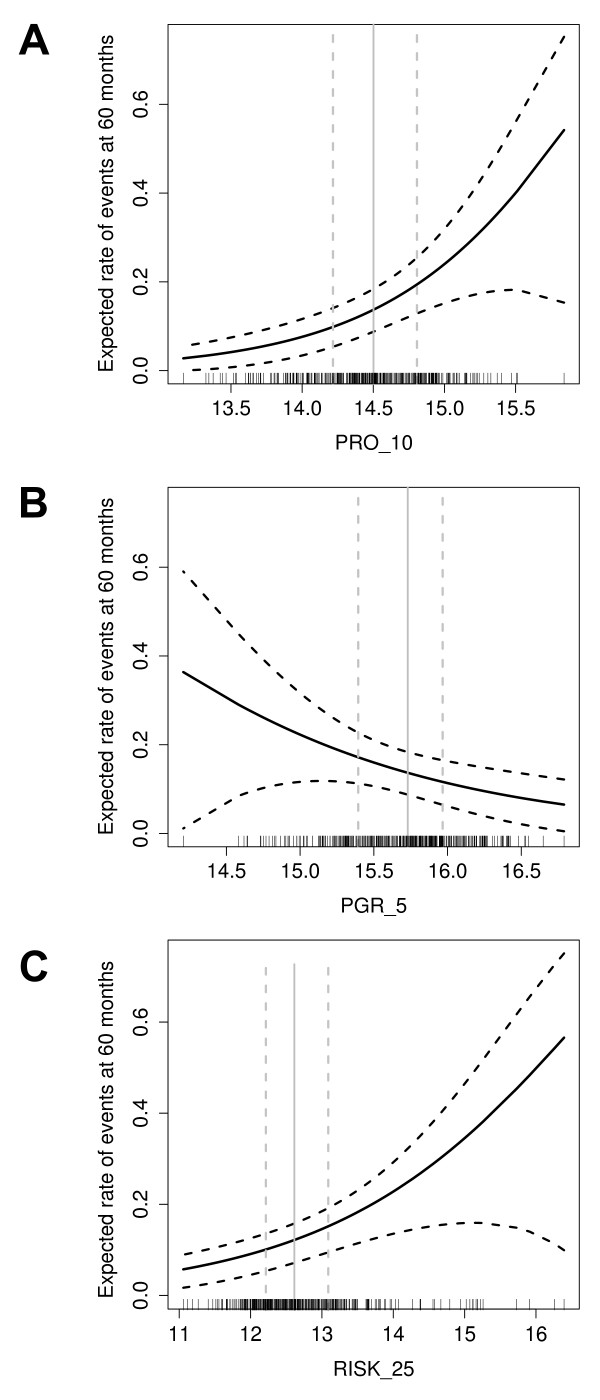
**Expected rate of disease-free survival (DFS)**. The expected rate of events at 60 months (solid line) is shown as a function of PRO_10 (A), PGR_5 (B) and RISK_25 scores (C). The 95% confidence intervals are indicated (dashed lines). Vertical lines represent the median of all scores (solid line) and 25% and 75% quantiles (dashed lines).

Figure 4 shows the estimated rate of recurrence as a function of PRO_10, PGR_5 and RISK_25 scores. The PRO_5, PRO_10 and the RISK_25 scores remained significant predictors of DFS when applied to patients with grade II breast cancer.

### PRO_10 and PGR_5 scores are independent risk factors in multivariate analyses

The impact of the molecular scores PRO_10 and PGR_5 was further documented in multivariate models comprising clinicopathologic predictors and molecular scores that were significant in univariate analyses.

Multivariate analyses revealed that PRO_10 is a predictor of DFS independent of tumor size (T), number of positive lymph nodes (N), grade (G) and Ki-67 LI. PRO_10 represents proliferation-related genes and it was of interest to compare it to Ki-67. Table 2 shows the results of multivariate analyses including T, N, G and either Ki-67 (model 1) or PRO_10 (model 3) in comparison with a model containing both markers (model 2). The full model (model 2) was significantly better than model 1 (LRT p = 0.0071). No significant difference was found for PRO_10 between models 2 and 3 (LRT p = 0.8075). Thus, adding PRO_10 to T, N, G and Ki-67 significantly improved the model. In contrast, adding Ki-67 to T, N, G and PRO_10 did not bring additional information.

The same procedure was used to evaluate whether PGR_5 further improved model 6 containing T, N, G and PRO_10 (Table 2). The full model including all 5 variables (model 5) performed better than model 4 (T, N, G, PGR_5; LRT p = 0.0089) and model 6 (T, N, G, PRO_10; LRT p = 0.0339). Both, PGR_5 and PRO_10 remained significant in model 5 suggesting that the two scores contain independent information with respect to prognosis and outcome.

## Discussion

Gene expression profilings define clinically relevant gene signatures [15,17,37,38]. For the present work, we selected genes correlating with the ER, PgR, Her2 and proliferative status using a meta-analysis of gene expression profiles [28]. The prognostic power of resulting gene expression scores for ER, PgR, proliferation and overall risk of recurrence was validated using tissues and clinical data from a representative subset of participants of trial BIG 1-98 confirming the correlation structure of these genes and their association with clinical and outcome variables.

Multiple genes representing each score were quantified by qRT-PCR. RNA from 82 matched FF and FFPE tissues were compared by qRT-PCR on TLDAs. The mean increase of raw Ct values between RNA from FF and FFPE tissues was 1.3 units. This is similar to the findings of Cronin and co-workers (+2.0 units) in a comparable setting [39]. Duration of formalin fixation, storage time and conditions influence the quality of RNA derived of FFPE tissues with direct effects on the sensitivity of subsequent PCR reactions [40]. However, normalization effectively compensated for this shift of Ct values (Additional File 2, Fig S1 and Additional File 3, Figure S2).

The mean expression of eight genes related to ER and five genes related to PgR were used to calculate the ER_8 and PGR_5 scores. Scores representing different functional categories were combined in RISK_25 score. The molecular scores determined from 82 paired samples of FF and FFPE tumors were highly concordant, as were molecular scores and immunohistochemically assessed parameters demonstrating the reliability of the procedure.

Molecular scores were validated in an independent set of tumor tissues from 342 participants of trial BIG 1-98. In contrast to histological analyses which can also be performed from tissue sections that contain considerable normal, stromal or fat components the architecture of the tissue is completely lost during work up for molecular analyses and therefore, it was important to exclude samples with inadequate tumor content. A histological section was taken from the immediate vicinity of each sample that was used for molecular analyses. Each section was assessed by an experienced pathologist (H.J.A.) and molecular analyses were restricted to samples containing at least 30% tumor cells. For comparison, RNA was also isolated from tumor-surrounding cells which led to rather poor RNA recoveries from comparable tissue areas (data not shown). However, this does not exclude that tumor-surrounding cells may have a limited impact on molecular scores in such analyses. Contamination by non-tumor cells may be reduced by macrodissecting tumors before RNA isolation and molecular assessment. The same procedure would also make tumors accessible to molecular analysis when sections contain less than 30% tumor cells.

Classification of patients by low and high PRO_10 and RISK_25 scores corresponded to low and high risk of recurrence. PRO, RISK and PGR scores were prognostic for DFS not only in the entire patient population but also in a subpopulation of patients with node positive disease (Figure 3D and 3E). We provide evidence independent of Genomic Health™ that a RISK score based on similar biological processes as the recurrence score (RS), but with other genes selected through a different procedure, can predict DFS [29,41,42]. In contrast to the RS which was validated with tamoxifen-treated patients, PRO_10, RISK_25 and PGR_5 scores were validated with patients treated with tamoxifen, letrozole or a sequence of both drugs; therefore, they may apply to patients who received either of these drugs.

Histological grading is an important factor in estimating the risk of recurrence of patients with breast cancer [2,43]. Recently, Sortiriou and colleagues have developed the gene expression grade index (GGI) based on the expression of 97 genes related to proliferation. They demonstrated that grade II cancers are comprised of tumors which are similar to genomic grade I or grade III with corresponding clinical outcomes [16,44]. Our findings agree with these observations as grade II tumors could be further classified into low and high risk of recurrence by 10 genes (PRO_10) (Figure 3C) or even by 5 genes (PRO_5 score) (data not shown). Seven of the PRO_10 and three of the PRO_5 genes are also part of GGI. The PRO_5 genes (Table 1) corresponded to the proliferation-related genes of the RS [29]. The assessment of gene signatures related to proliferation such as GGI or PRO scores is of special interest in ER positive, grade II breast cancer for whom therapeutic decisions are often difficult. Both, GGI and RS were shown to be associated with response to chemotherapy [45,46]. In contrast to GGI which requires FF tumor material, PRO scores or RS can be determined from a few microtome slices or cores such as used for tissue microarrays [47]. Material for molecular analysis can be taken from the same FFPE tissue block used for histological and immunohistochemical analyses without interfering with clinicopathological workflow.

The prognostic value of Ki-67 in early breast cancer was recently confirmed [48]. However, Ki-67 is not used uniformly in clinical practice [49,50] as it appears to be difficult to agree on cut-off values separating high and low proliferation tumors or on its value in assisting the choice of adjuvant therapy [50,51]. Therefore, instead of dichotomizing Ki-67 it may be more feasible to use Ki-67 as continuous variable [52]. Here, we made a comparison between centrally assessed Ki-67 LI and a qRT-PCR based proliferation signature. The PRO_10 score correlated with Ki-67 LI, and both were significant predictors of DFS in univariate Cox analyses. In multivariate models however, PRO_10 offered superior prognostic value and outperformed Ki-67 LI (Table 3). Moreover, the PRO_10 score added independent prognostic information to anatomical staging.

**Table 3:** Cox Proportional Hazard Analyses.

Covariate	P- value	HR (95% CI)
Univariate Analyses*		

Clinicopathological Variables		
HER2	0.7816	1.18 (0.36 - 3.84)
PgR	0.5147	0.78 (0.36 - 1.66)
Histological grade	0.0032	1.99 (1.26 - 3.14)
Ki-67 LI	0.0226	1.02 (1.00 - 1.04)
Tumor size	0.0047	1.22 (1.06 - 1.39)
Number of positive nodes	<0.0001	1.13 (1.08 - 1.18)
Treatment (4 categories)	0.1540	-
Molecular scores		
HER2_2	0.1080	1.20 (0.96 - 1.51)
PGR_5	0.0344	0.66 (0.44 - 0.97)
PRO_5	0.0003	2.14 (1.42 - 3.22)
PRO_10	<0.0001	2.09 (1.45 - 3.00)
RISK_25	0.0001	1.54 (1.24 - 1.91)

Multivariate Analyses: Comparison of PRO_10 and Ki-67 LI**		

**Model 1: **log-likelihood = -179.38, Deviance = 188.11		
Number of positive nodes	<0.0001	1.19 (1.12 - 1.27)
Tumor size	0.0370	1.19 (1.01 - 1.39)
Grade	0.4200	1.25 (0.72 - 2.17)
Ki-67 LI	0.1300	1.02 (1.00 - 1.04)
**Model 2: **log-likelihood = -175.75, Deviance = 180.71		
Number of positive nodes	<0.0001	1.19 (1.12 - 1.27)
Tumor size	0.1300	1.14 (0.96 - 1.34)
Grade	0.9600	0.99 (0.55 - 1.76)
PRO_10	0.0092	2.12 (1.20 - 3.72)
Ki-67 LI	0.8100	1.00 (0.97 - 1.03)
**Model 3: **log-likelihood = -175.78, Deviance = 180.77		
Number of positive nodes	<0.0001	1.19 (1.12 - 1.27)
Tumor size	0.1200	1.14 (0.97 - 1.34)
Grade	0.9400	0.98 (0.55 - 1.74)
PRO_10	0.0026	2.03 (1.28 - 3.23)

Multivariate Analyses: Role of PGR_5***		

**Model 4: **log-likelihood = -215.27, Deviance = 214.30		
Number of positive nodes	<0.0001	1.12 (1.07 - 1.16)
Tumor size	0.2000	1.11 (0.95 - 1.30)
Grade	0.0170	1.78 (1.11 - 2.87)
PGR_5	0.0570	0.68 (0.45 - 1.01)
**Model 5: **log-likelihood = -211.85, Deviance = 208.03		
Number of positive nodes	<0.0001	1.06 (1.06 - 1.16)
Tumor size	0.4300	1.07 (0.91 - 1.26)
Grade	0.3000	1.32 (0.78 - 2.23)
PRO_10	0.0092	1.73 (1.15 - 2.62)
PGR_5	0.0360	0.65 (0.43 - 0.97)
**Model 6: **log-likelihood = -214.10, Deviance = 211.25		
Number of positive nodes	<0.0001	1.11 (1.06 - 1.16)
Tumor size	0.1700	1.13 (0.95 - 1.34)
Grade	0.2100	1.40 (0.83 - 2.37)
PRO_10	0.0150	1.71 (1.11 - 2.62)

*Histological grading was analyzed according to three categories (histological grade I, II or III). Number of lymph node metastases and tumor size were continuous variables. PgR and Her2 were centrally assessed and binary IHC data were included in the analyses [30,31]. Centrally assessed Ki-67 labeling index and molecular scores were included as continuous variables.

**Data of 299 patients with available Ki-67 LI were included in model 1, 2 and 3, respectively.

***Data of all 342 patients were included in model 4, 5 and 6, respectively.

Abbreviations: HR, hazard ratio; CI, confidence interval; LRT, likelihood ratio test; Ki-67 LI, Ki-67 labeling index.

Models 3 and 6 should not be compared directly as they were fitted on different sample sizes, due to missing data in Ki-67 LI.

PgR, as measured by immunohistochemistry [30] or microarray analysis [53], was shown to positively correlate with prognosis. Here we show that the molecular PGR_5 score was also positively associated with DFS (Figure 4) and added independent prognostic information to anatomical staging and PRO_10 (Table 3). Thus, PGR_5 and PRO_10 scores independently predict prognosis in the BIG 1-98 population.

Compared to immunohistochemically assessed parameters, qRT-PCR based scores are quantitative, relatively independent on operator expertise and less affected by inter-observer variability. The procedure is simple, economical and can be standardized easily with good control genes, reference samples and quality control procedures.

The results of this study are based on a limited number of patients and follow-up time (60 months). Similar analyses with independent, larger sample sizes and more mature follow-up data are planned to further consolidate the prognostic and possibly predictive value of the proposed scores in each treatment arm separately.

Gene expression profiling has improved the understanding of molecular subtypes of breast cancer. FFPE material is not widely used although it may facilitate and speed up the development and validation of novel gene signatures due to the availability of well-characterized tissues from numerous clinical trials [54,55]. The same material can be used for molecular diagnostics. The investigation of gene signatures may become more important in the future as an increasing proportion of agents under development for breast cancer treatment have defined molecular targets. Early integration of biomarker analysis in the drug development process has the potential to improve the specificity and efficiency of novel therapeutics. This opens the possibility to further individualize therapy of patients with breast cancer.

## Conclusions

We define four molecular scores based on quantitative measurement of gene expression with RNA derived of FFPE tissues. The genes for each score were selected from a large meta-analysis of microarrays. The genes do not coincide with genes used for other molecular scores like the RS (except genes that were previously used as immunohistochemical markers such as ER, PgR or Her2). Two of the described scores are shown to be independent predictors of disease-free survival of postmenopausal patients with operable, estrogen receptor positive breast cancer. The proliferation-associated score outperforms the Ki-67 labeling index measured by immunohistochemistry.

## List of abbreviations

AUC: area under the (ROC) curve; CI: confidence interval; DFS: disease-free survival; ER: estrogen receptor; FF: fresh frozen; FFPE: formalin-fixed, paraffin embedded; HR: hazard ratio; IHC: immunohistochemistry; GGI: gene expression grade index; LI: labeling index; LRT: likelihood ratio tests; PCR: polymerase chain reaction; RIN: RNA integrity number; PgR: progesterone receptor; ROC: receiver operating characteristic; RS: recurrence score; TLDA: TaqMan Low Density Arrays.

## Competing interests

JA, VP, MD, PW, AB, AO, AGH, GV, HJA, SA and RJ declare that they have no competing interest. B.T. holds stocks from Novartis (Ciba Geigy) since 1990.

## Authors' contributions

JA, SA and RJ organized the study, planned the experiments and wrote the manuscript. SA and BT organized samples from the International Breast Cancer Study Group. AO and AB carried out RNA isolations, quality controls and gene expression measurements. VP, PW, MD and AGH carried out the statistical analyses. HJA and GV were responsible for histological assessment of stained sections. All authors contributed to the manuscript, they read and approved the final manuscript.

## Pre-publication history

The pre-publication history for this paper can be accessed here:

http://www.biomedcentral.com/1471-2407/10/37/prepub

## Supplementary Material

Additional file 1 Publicly available gene expression data from breast cancer studies. Click here for file

Additional file 2 **Effect of normalization**. Mean expression of 34 assays determined for 82 RNAs isolated from FFPE and from corresponding FF tissue. Shown are the differences between FFPE and FF before (Raw) and after normalization against the mean of three control genes (UBB, RPLP0 and GUSB) (Normalized). Click here for file

Additional file 3 **Unsupervised hierarchical clustering of data from FF- and FFPE-derived RNA**. Shown are heat maps based on normalized expression from RNA of FF (A) and FFPE tissues (B). Proliferation (red box), Her2 (blue box) and ER or PgR related genes (green box) are indicated. The hormone receptor status of each tumor was also assessed by IHC. ER negative (closed circles) and Her2 positive tumors (open circles) are indicated. Click here for file

Additional file 4 **Distribution of molecular scores**. Shown are histograms of ER, PGR and HER2 scores and fitted mixtures of Gaussian distributions. Results of 82 matched samples are shown for ER_8 (A, B), PGR_5 (C, D) and HER2_2 (E, F) scores derived from FF (A, C, E) and FFPE tissues (B, D, F). Click here for file
