# Supplementary material for: Molecular risk assessment of BIG 1-98 participants by expression profiling using RNA from archival tissue
Source: BMC Cancer. 2010 Feb 9;10:37. doi: 10.1186/1471-2407-10-37 (PMC2829498; doi:10.1186/1471-2407-10-37)
Supplement: Additional file 1 — Publicly available gene expression data from breast cancer studies. [file 1471-2407-10-37-S1.PDF]

Table S1: Publicly available gene expression data from breast cancer studies

Dataset symbol	Number of arrays	Institution	Reference(s)*	Platform	Data source	Number of GeneIDs
NKI	337	Nederlands Kanker Instituut (Amsterdam, The Netherlands)	[19,20]	Agilent	Author's website	13,120
EMC	286	Erasmus Medical Center (Rotterdam, The Netherlands)	[21]	Affymetrix U133A	GEO: GSE2034	11,837
UPP	249	Karolinksa Institute (Uppsala, Sweden)	[3,11]	Affymetrix U133A,B	GEO: GSE4922	15,684
STOCK	159	Karolinska Institute (Stockholm, Sweden)	[3,13]	Affymetrix U133A,B	GEO: GSE1456	15,684
DUKE	171	Duke University (Durham, NC, USA)	[8]	Affymetrix U95Av2	Author's website	8,149
UCSF	161 + 8	University of California at San Francisco (USA)	[9]	cDNA	Author's website	6,178
UNC	143 + 10	University of North Carolina (Chapel Hill, NC, USA)	[7]	Agilent HuA1	Author's website	13,784
NCH	135	Nottingham City Hospital (Nottingham, UK)	[12]	Agilent HuA1	AE: E-UCON-1	13,784
STNO	115 + 7	(Oslo, Norway)	[16]	cDNA	Author's website	5,614
JRH1	99	John Radcliffe Hospital (Oxford, UK)	[17]	cDNA	Journal's website	4,112
JRH2	61	John Radcliffe Hospital	[18]	Affymetrix U133A	GEO: GSE2990	11,837
MGH	60	Massachusetts General Hospital (Boston, MA, USA)	[10]	Agilent	GEO: GSE1379	11,421
expO	239	International Genomic Consortium	[41]	Affymetrix U133v2	GEO: GSE2109	16,634
TGIF1	49	EORTC trial 10994	[5]	Affymetrix U133A	GEO: GSE1561	11,837
BWH	40 + 7	Brigham and Women's Hospital (Boston, MA, USA)	[14]	Affymetrix U133v2	GEO: GSE3744	16,634
TRANSBIG	253	TRANSBIG Consortium	[2]	Agilent	AE: E-TABM-77	1,052
EMC2	180	Erasmus Medical Center	[6]	Affymetrix (custom)	GSE3453	86
HPAZ	96	Hospital La Paz (Madrid, Spain)	[4]	RT-PCR	Appendix of [4]	61
Total		2,865 = 2,833 carcinomas + 32 nonmalignant breast tissues		Number of the union of all GeneIDs:		17,198
				Number of GeneIDs common to genomic platforms:		1,963

Datasets UNC, STNO, UCSF, and BWH include a small number of normal breast or fibroadenoma tissue samples. AE, ArrayExpress (accession); Affymetrix, Affymetrix, Inc., Santa Clara, CA, USA; Agilent, Agilent Technologies, Inc., Santa Clara, CA, USA; EORTC, European Organization for Research and Treatment of Cancer; GEO, Gene Expression Omnibus (accession); RT-PCR, reverse transcription-polymerase chain reaction.

Wirapati *et al.* *Breast Cancer Research* 2008 **10**:R65 doi:10.1186/bcr2124

* References in Wirapati et al.

available at
http://breast-cancer-research.com/content/10/4/R65/table/T1
