# Supplementary figures and images for: Molecular risk assessment of BIG 1-98 participants by expression profiling using RNA from archival tissue
Source: BMC Cancer. 2010 Feb 9;10:37. doi: 10.1186/1471-2407-10-37 (PMC2829498; doi:10.1186/1471-2407-10-37)

Fig S1 Effect of normalization.

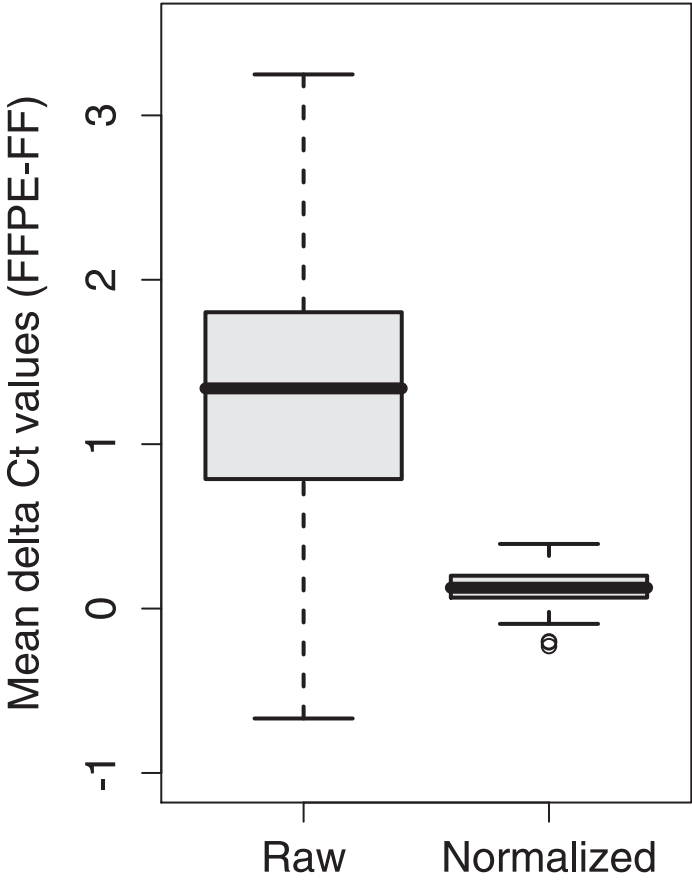

Supplement: Additional file 2 — Effect of normalization. Mean expression of 34 assays determined for 82 RNAs isolated from FFPE and from corresponding FF tissue. Shown are the differences between FFPE and FF before (Raw) and after normalization against the mean of three control genes (UBB, RPLP0 and GUSB) (Normalized). [file 1471-2407-10-37-S2.PDF]

Fig S2 Unsupervised hierarchical clustering of data from FF- and FFPE-derived RNA.

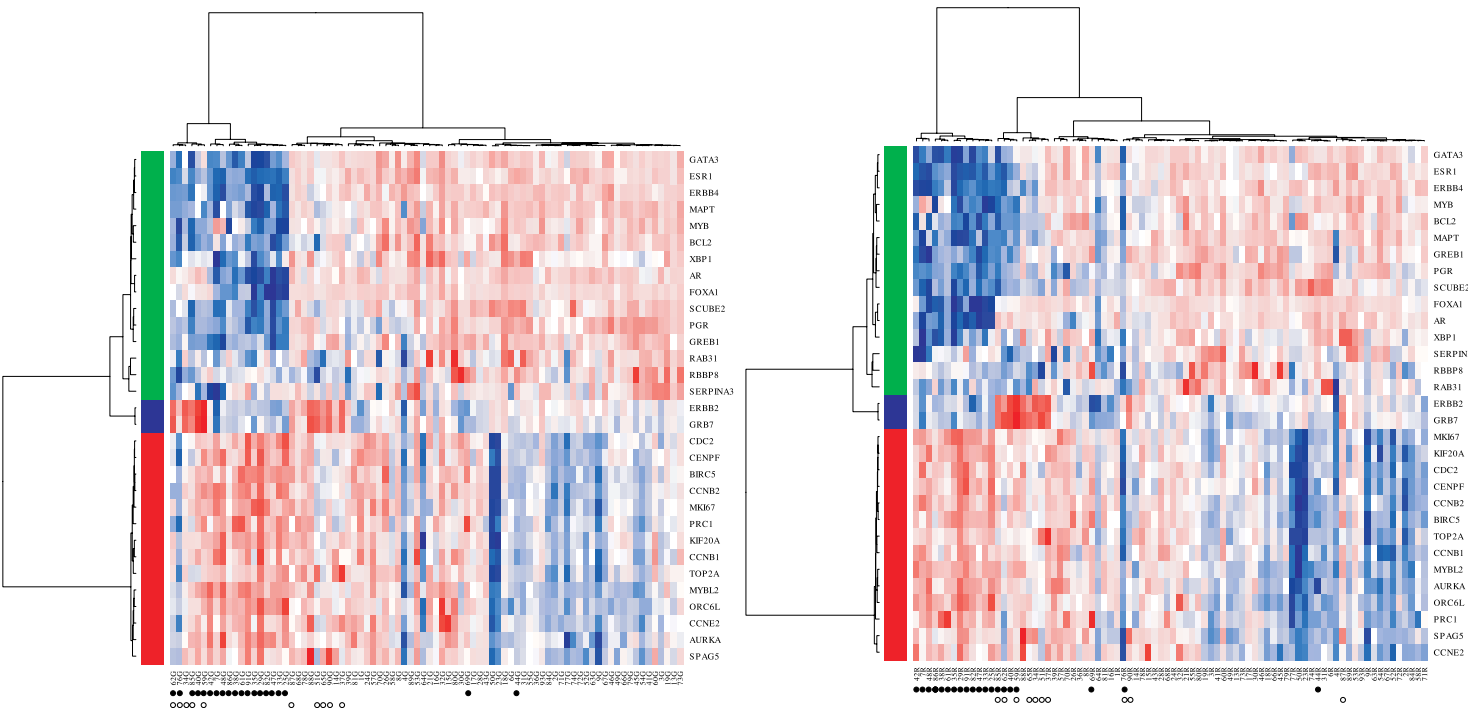

Supplement: Additional file 3 — Unsupervised hierarchical clustering of data from FF- and FFPE-derived RNA. Shown are heat maps based on normalized expression from RNA of FF (A) and FFPE tissues (B). Proliferation (red box), Her2 (blue box) and ER or PgR related genes (green box) are indicated. The hormone receptor status of each tumor was also assessed by IHC. ER negative (closed circles) and Her2 positive tumors (open circles) are indicated. [file 1471-2407-10-37-S3.PDF]

Fig S3 Distribution of molecular scores.

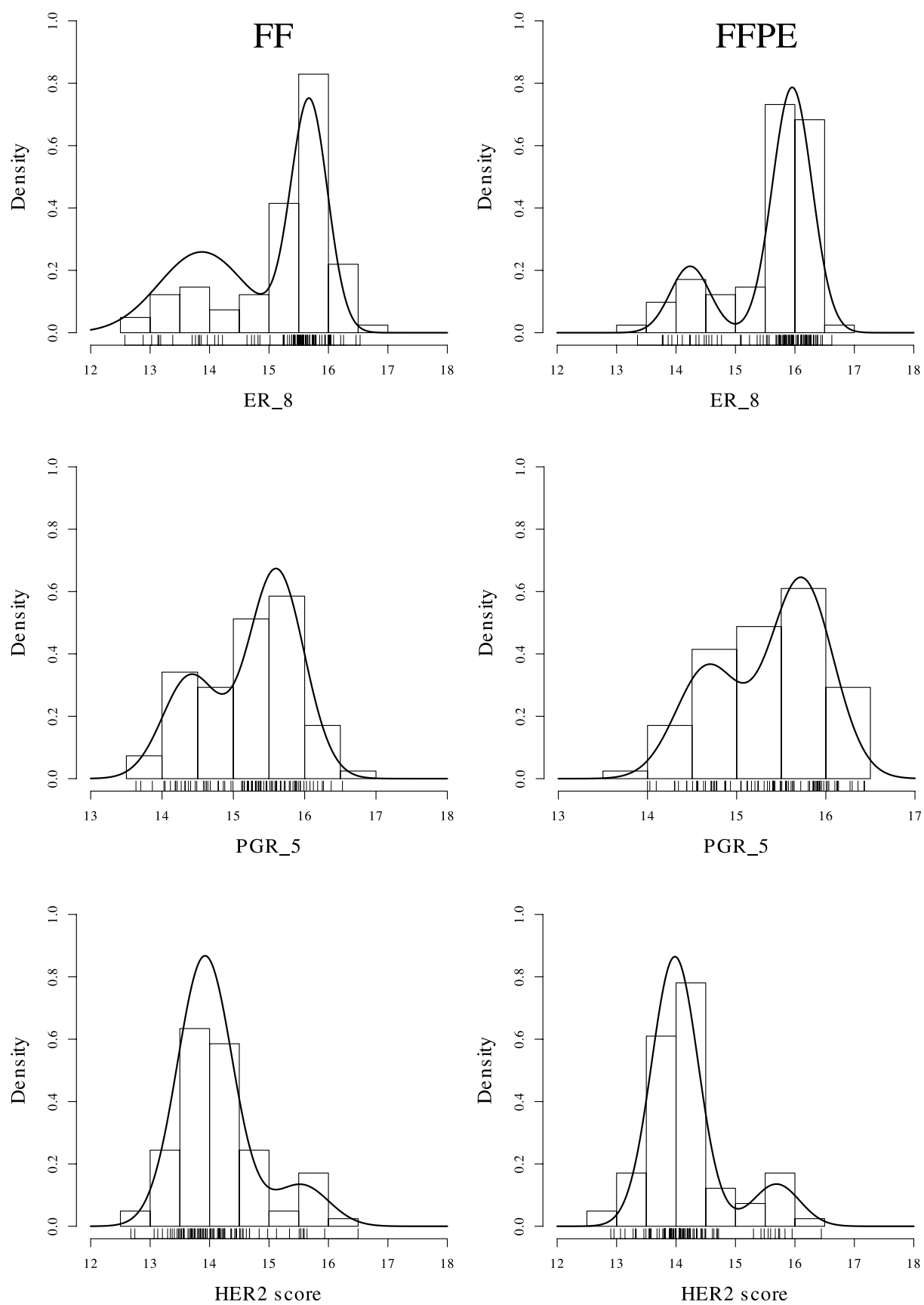

Supplement: Additional file 4 — Distribution of molecular scores. Shown are histograms of ER, PGR and HER2 scores and fitted mixtures of Gaussian distributions. Results of 82 matched samples are shown for ER_8 (A, B), PGR_5 (C, D) and HER2_2 (E, F) scores derived from FF (A, C, E) and FFPE tissues (B, D, F). [file 1471-2407-10-37-S4.PDF]
